# Pathologist’s perspectives on reporting of tumour budding in colorectal cancer

**DOI:** 10.3332/ecancer.2021.1337

**Published:** 2021-12-23

**Authors:** Paromita Roya, Lateef Zameer, Debdeep Dey, Jayati Datta, Anand Bardia, Deepak Mishra

**Affiliations:** Tata Medical Center, Department of Pathology, 14 MAR (E-W), Newtown, Kolkata, West Bengal 700156, India; a https://orcid.org/0000-0003-0379-5999

**Keywords:** tumour budding, colon cancer, ITBCC (International Tumor Budding Consensus Conference)

## Abstract

**Purpose:**

Tumour budding (TB) is an important adverse prognostic factor in colon cancer, which can also guide adjuvant treatment in stage II colorectal carcinoma. The International Tumor Budding Consensus Conference (ITBCC) recommended a three-tiered scoring system to streamline the scoring of budding across the globe. The goal of this survey is to understand the variation in reporting practice, globally.

**Methods:**

A short survey was designed as an online questionnaire and shared via social media platforms and emails to pathology society groups in various countries.

**Results:**

A majority of the 294 respondents (186/294; 63.3%) report budding in colorectal carcinoma. This figure differed significantly from 53.4% in Asia to 97.4% in North America. The most common (56.4%) reason for not reporting TB was because it is yet not a mandatory recommendation in the various datasets (e.g. The College of American Pathologists). The majority (78.9%) of the people who were reporting TB, used the ITBCC scoring system (scoring on a single hotspot 20× field). Most used 10× objective for screening (88.7%) and scored only at the invasive front (88.7%). Immunohistochemistry (8.6%) or deeper cuts (24.2%) were rarely used. TB scoring took 10 minutes or less in most (87.1%).

**Conclusion:**

Though budding is well accepted among specialist gastrointestinal pathologists, it is still not universally accepted as an important prognostic parameter across the globe. The hesitancy for reporting is due to a combination of lack of clinical demand and extra effort and time involved in counting the ITBCC score.

## Background/introduction

Tumour buds are defined as small tumour cell clusters of up to four cells, seen at the invasive front of cancer. Multiple studies have shown that tumour buds connote poor prognosis and are an independent predictor for metastasis in colorectal cancer (CRC). The College of American Pathologists (CAP) has recommended reporting this parameter in colon specimens including in polypectomies for malignant polyps, using the three-tiered scoring system recommended by the International Tumor Budding Consensus Conference (ITBCC). The National Comprehensive Cancer Network (NCCN) has also recently included tumour budding (TB) as an adverse prognostic factor for stage II CRC, to guide adjuvant treatment [1–3]. Dawson *et al* [4] suggested adjuvant chemotherapy in stage II CRC with a high score (>10 buds/20× high power field of 0.785 mm^2^) budding and at least one more adverse prognostic marker. A large multicentre prospective trial (SACURA trial) has also shown a recurrence-free survival benefit of adjuvant chemotherapy in stage II CRC with TB [5]. Though the clinical significance of TB is well-established, there is still a lack of uniformity in reporting TB in colon cancer. We planned a practice survey to understand how commonly TB is being reported in CRC, the reasons, challenges and methods used to gain a better understanding of prevalent practice globally.

## Methods

The survey was designed as an online questionnaire using a free form creation app (Google Forms). There were 13 questions that covered the elements of practice (geography, academic or service, specialist or generalist), reasons for or against reporting TB, the methods used, the time spent and the perceived clinical relevance in routine practice. All entries were anonymous. The link was shared via social media platforms and emails through various pathology societies and groups.

## Results

There were a total of 294 respondents to the survey from 25 countries. The details of the respondents are given in Table 1. We lacked participants from China, Japan and other countries in the far east. Twenty-six participants did not mention their country of service. The respondents were equally distributed in academic or clinical service, and about a third were specialist gastrointestinal (GI) pathologists. The annual caseloads were varied, but >72% reported over 50 CRC cases annually.

The majority of respondents (186/294; 63.3%) declared that they report TB in some or all of their cases. Of them, 103/294 (35%) reported budding in all cases, while 59/294 (20.1%) reported only in stage I or II cancers and 24/294 (8.2%) reported only in polypectomy/endoscopic mucosal resection (EMR) specimens.

While a slightly higher proportion of respondents from academic practice reported budding compared to those in service-based practice, the difference was not statistically significant (67.9% versus 59.2%, *p* = 0.125). On the other hand, a significantly higher proportion of pathologists in specialist GI pathology practice reported budding, compared to those who were not (84.8% versus 52.3%, *p* < 0.001). The geographic distribution of respondents reporting budding was not uniform. The proportion reporting TB from Asia (53.4%), comprising mainly respondents from India, was much lower than that reported from North America (97.4%), Europe (81.5%), South America (100%), Oceania (100%) and Africa (75%). Of the pathologists who were reporting TB, 55 (29.4%) confirmed it was used to decide adjuvant treatment, while 92 (50%) were not sure if clinicians were using it.

The main reasons for or against reporting TB in clinical practice are enumerated in Table 1 (second part). The majority (100/108, 92.59%) of the participants who were not reporting budding justified that this was still an optional criterion and that they would start when it was made mandatory by CAP or other dataset guidelines.

There was some heterogeneity in the method of reporting TB, as depicted in Table 1. The majority of respondents used the ITBCC score groups using the 20× objective and a field size of 0.785 mm^2^. The 10× objective (followed by 4×) was used most commonly to scan the slides to select the worst hotspot for counting TB. Only a small number of pathologists used deeper sections or immunohistochemistry (IHC) to aid counting. The majority (87.1%) of respondents felt that reporting TB took less than 10 minutes of their time.

## Discussion

To the authors’ knowledge, this is the first survey looking at reporting practices of TB internationally. One of the factors limiting universal adoption of ITBCC scoring of TB is the ambiguity surrounding the impact of this novel feature on treatment decisions. There is also a perception that the count may be tedious to do with potential inter and intra-observer variability and some participants used a present/absent method of reporting TB rather than doing an actual count or following ITBCC guidelines.

The lack of easy availability of 20× high power objectives in resource-constrained settings is a valid limitation to adopting the ITBCC scoring system. In a prior publication in our centre, we have developed and validated a method of using a 40× objective for scoring in countries that do not commonly use a 20× objective and demonstrated high interobserver concordance and significant correlation with prognosis using both methods (20× and 40× score) of reporting TB [6]. This was the first such paper reporting on technique and validation in an Indian patient cohort in CRC. However, whether the 40× scoring required more time compared to the 20× score, was not evaluated.

To keep the survey short, we did not elaborate on all the details regarding reporting TB. One of the limitations of this survey is that the number of pathologists reporting TB in both EMR and stage I/II cases was not captured. Another limitation is that respondents from other countries were more likely to be specialist GI pathologists and in academic practice and reflects the bias in dissemination and response to the survey. The experience of the pathologist and familiarity with the concept of TB was not captured in this survey and we cannot determine if this had an impact on reporting patterns. As in any survey, recall bias may have impacted the answers to some questions, including the time taken for TB scoring.

Some pathologists were using IHC to identify buds but reporting TB score on Hematoxylin-eosin (HE). This option was not included in the survey options. Reporting of TB in neoadjuvant-treated CRC specimens, colon biopsies, pT1 CRCs and other (non-CRC) site groups was not addressed by our survey. The issue of differentiating TB from poorly differentiated tumour cell clusters was also not addressed.

Some of the comments we received from the participants are – that oncologists were still not aware of the prognostic and predictive significance of TB, even though it has been recommended in commonly used treatment practice guidelines like the NCCN and are more focused on the molecular characteristics or microsatellite instability (MSI) status to guide treatment. These tests are however expensive and universal screening for MSI in CRC is still not practised in large parts of the world.

## Conclusion

Our survey gives us a real-world picture of the patterns of adoption of reporting TB in CRC. It also gives us an insight into the causes of the variation in reporting styles among the western population (mainly the USA and Canada) and specialist GI pathologists, versus those in Southeast Asia (India). It highlights the need for more clinical evidence on the impact of TB on prognosis and adjuvant treatment approaches in different stages of CRC.

## List of abbreviations

TB, Tumour budding; CRC, Colorectal cancer; ITBCC, International Tumor Budding Consensus Conference

## Declarations

### Funding

No external funding was sought for this study.

### Conflicts of interest

There are no conflicts of interest of any of the authors in this study.

### Availability of data/material

Available.

### Code availability

Not applicable.

### Authors’ contributions

All the authors were involved in the study design, questionnaire preparation, survey dissemination and final manuscript write-up. The first author was additionally involved in the conceptualisation and statistical analysis of the results of the survey.

### Ethics approval

Not applicable.

### Consent to participate

Not applicable.

### Consent for publication

Not applicable.

## Figures and Tables

**Table 1.** Survey results.

Demographic details of respondents			
**Characteristics**	**n (%)**	**Percentage of pathologists reporting TM**	**p value**
**Country/continent**			*p* < 0.000
India	185 (69%)	54.6%	
North America	38 (14.2%)	97.4%	
Europe	22 (8.2%)	81.5%	
South America	4 (1.5%)	100%	
Oceania	4 (1.5%)	100%	
Others	15 (5.5%)		
**Types of practice**			*p* = 0.125
Primarily academic	137 (46.6%)	67.9%	
Primarily clinical	157 (53.4%)	59.2%	
**Specialisation**			*p* < 0.000
Specialist GI pathologists	99 (33.7%)	84.8%	
General pathologists	195 (66.3%)	52.3%	
**Annual caseload of CCs**			*p* < 0.001
<50 cases	80 (27.2%)	38.8%	
50–100 cases	88 (29.9%)	55.7%	
100–200 cases	57 (19.4%)	73.7%	
>200 cases	66 (22.4%)	78.8%	
			
**Reasons given for reporting or not reporting TB**			
Reasons	*n* (%)		
Not reporting TB	108		
It is still not a mandatory reporting criteria in the CAP or Royal College of Pathologists (RCPath) datasets	59 (54.6%)		
It is not used for making adjuvant treatment decisions	42 (38.8%)		
The method is too tedious	18(16.6%)		
The evidence for the prognostic significance of this parameter is not robust enough	17 (15.7%)		
Lack of availability of a 20× objective in resource-constrained setting	12 (11.1%)		
			
Reporting TB	186		
It is a recommended reporting parameter in CAP/RCPath protocol	108 (58 %)		
It is a significant risk factor for nodal metastasis and prognosis	100 (53.7 %)		
It is used for making treatment decisions for adjuvant treatment for stage II cancer (in conjunction with other factors)	65 (34.9 %)		
All the above three	3 (1.6 %)		
It is useful for treatment decision in EMR specimens/malignant polypectomies	5 (2.6 %)		
**Characteristics of TB reporting**			
Characteristics	*n* (%)		
**Method of reporting**	181		
ITBCC score groups – low/intermediate/high by counting on 20× hotspot field with a field size of 0.785 mm^2^	123 (67.9%)		
ITBCC score groups (in 20× hotspot field with a field size of 0.95 mm^2^)	20 (11%)		
ITBCC score groups and absolute bud count	32 (17.7%)		
Presence or absence	1 (0.5%)		
Not scored but reported as a poorly differentiated component	1 (0.5%)		
Others	4 (2.2%)		
**Objective used**	184		
10×	71 (38.6%)		
2.5×	4 (2.2%)		
20×	36 (19.6%)		
40×	14 (7.6%)		
4×	59 (32%)		
**Location of tumour buds for counting**	71		
At the invasive front of tumour only	63 (88.7%)		
At the tumour front or core without differentiating between the two	6 (8.5%)		
Giving separate counts for tumour front and core	2 (2.8%)		
**Deeper sections used**	186		
Yes	45 (24.2%)		
**IHC used**	186		
Yes	16 (8.6%)		
**Time taken to report TB**	186		
<5 minutes	90 (48.4%)		
5–10 minutes	72 (38.7%)		
10–15 minutes	21 (11.3%)		
>15 minutes	3 (1.6%)		
